# Safety protocols in an exercise facility result in no detectable sars‐CoV2 spread: A case study

**DOI:** 10.14814/phy2.14967

**Published:** 2021-07-21

**Authors:** Burak T. Cilhoroz, Lara R. DeRuisseau

**Affiliations:** ^1^ Department of Exercise Sciences Syracuse University Syracuse NY USA; ^2^ Department of Biological Sciences Le Moyne College Syracuse NY USA

**Keywords:** COVID‐19, exercise, safety protocols

## Abstract

Coronavirus 2019 (COVID‐19) disease has been a public health emergency of international concern with millions of confirmed cases globally. Closed environments with reduced ventilation contribute to the spread of COVID‐19, including superspreading events. Exercising in closed places further increases the risk for transmission. Therefore, many fitness facilities were closed as part of mandated shutdowns early in the pandemic. Evidence‐based safety protocols have now emerged and substantially reduce the risk of transmission. We report three positive cases of SARS‐CoV‐2 identified at a Dojo exercise facility in Manlius, NY, at three distinct time points. All cases were present in the Dojo 2 days prior to symptoms, a time period considered to be highly infectious. The safety protocols included universal mask wearing (no valves), multiple high‐efficiency particulate air (HEPA) filters, and reduced capacity which resulted in no known spread of COVID‐19.

## Graphical abstract

Over a time period between October 2020 and December 2020, three different cases (one teen, one adult, and one child) were identified in a Dojo in Manlius, NY with safety protocols in place. The positive cases occurred without overlap and there was no indication of virus transmission in the exercise facility. More importantly, all three individuals with positive results were in the Dojo practicing 2 days before the occurrence of symptoms, a time period of peak viral load of SARS‐CoV‐2, suggesting the masks, increased ventilation, and social distancing implemented were successful.

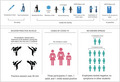

## 1 INTRODUCTION

Currently, the number of COVID‐19 cases has approached 170 million with approximately 3.5 million deaths worldwide (WHO, 2020). SARS‐CoV‐2, the virus leading to COVID‐19 disease has a reproduction number (i.e., transmission potential for a disease) of 2.0–2.5, higher than those of similar viruses, SARS‐CoV and Middle East respiratory syndrome, suggesting that SARS‐CoV‐2 is more transmissible (Zhao et al., 2020). Ways to mitigate SARS‐CoV‐2 spread while safely opening businesses and maintaining physical and mental wellness are of primary importance for communities.

Exercise facilities represent a business that offers social, emotional, and physical activities. They are vital to many communities, by improving well‐being and promoting inclusion (Higgerson et al., 2018). Exercise facilities have been last to re‐open in multiple cities around the world. However, they may pose even higher risk for the virus transmission than typical indoor work settings. Indeed, an early study indicated that a nationwide dance class in fitness facilities triggered over 110 COVID‐19 cases in Korea (Jang et al., 2020). Three COVID‐19 clusters were also identified in exercise facilities in Japan by March 31 (Amagasa et al., 2020). The first cluster reported in an exercise facility included 25 primary cases of COVID‐19 that spread to 14 additional individuals in the same facility, which is considered the second highest infection rate in Japan as of March 21 (Amagasa et al., 2020). Moreover, Lendacki et al., (2021) revealed a COVID‐19 outbreak at a Chicago exercise facility where 68% (55/81) of participants practicing in‐person exercise classes between August 24 and September 1 contracted the virus. Participants had to wear a mask, have their temperature taken, and symptoms were monitored on entry (Lendacki et al., 2021). However, most of them did not follow the requirement of mask use during exercise and room ventilation was not assessed in the study (Lendacki et al., 2021). Groves et al. (2021) reported a cluster of COVID‐19 cases in three Hawaiian exercise facilities (with some similar instructors) where 45% (21/47) of participants practicing stationary cycling, personal training, and/or kickboxing classes between June 27 and July 1 contracted the virus. The instructor(s) who later tested positive, shouted for encouragement and training. Social distancing was followed in most, but not all classes, and most participants did not wear a mask and no HEPA filters were in use. These findings reinforce the high transmission rate of SARS‐CoV‐2 in exercise facilities when safety protocols are not appropriately followed or no safety protocols are in place. Therefore, indoor exercise facilities may pose a particularly high risk for infection during virus outbreaks such as the COVID‐19 pandemic if no formal policies are followed to reduce transmission.

Evidence‐based guidelines should be present for exercise facilities to ensure the safety of their employees, exercising customers, and the general community. Providing an open‐air environment for exercising individuals can have considerable impact on restraining the transmission of COVID‐19 disease. In their preprint, Nishiura et al., (2020) also reported the likelihood of COVID‐19 was ~19 times higher in enclosed places than in open‐air environments. Poor ventilation can lead virus‐containing aerosol particles to stay concentrated and active for a longer time period and thus increase the risk of infection (Andrade et al., 2018). Maintaining social distance ≥6 feet at all times during practice is also important, but it is more effective when combined with mask wearing and proper ventilation in the facility. If an open environment exercise space is not possible then a proper ventilation protocol (using air cleaners/purifiers) and social distancing rules (≥6 ft apart) should be ensured. Additionally, Moriyama et al., (2020) showed the humidity of 50%–80% in the air at a room temperature of 22º C confers benefits for reducing the risk of infection.

The volume of ventilation can exceed 100 L/min during high intensity exercise (i.e., 80%–100% of VO_2peak_) (Wackerhage et al., 2020), potentiating the spread of the virus even faster than at rest and light (40%–60% of VO_2peak_), and moderate (60%–80% of VO_2peak)_ exercise. In some exercise classes shouting also occurs, which could increase respiratory droplets. Therefore, exercising individuals in a closed environment would need additional safety precautions to prevent COVID‐19 transmission. Face coverings, social distancing, reduced capacity, and additional ventilation/air filtering have been utilized in many environments, including gyms and other exercise facilities. It is unknown how common the spread is in these facilities when safety protocols are in place. Since the benefit of social exercise can be a vital component to an individual's overall wellness (Higgerson et al., 2018), it is important to determine if these environments can create a lower risk of COVID‐19 transmission with safety protocols.

## 2 CASE PRESENTATION

**FIGURE 1:**
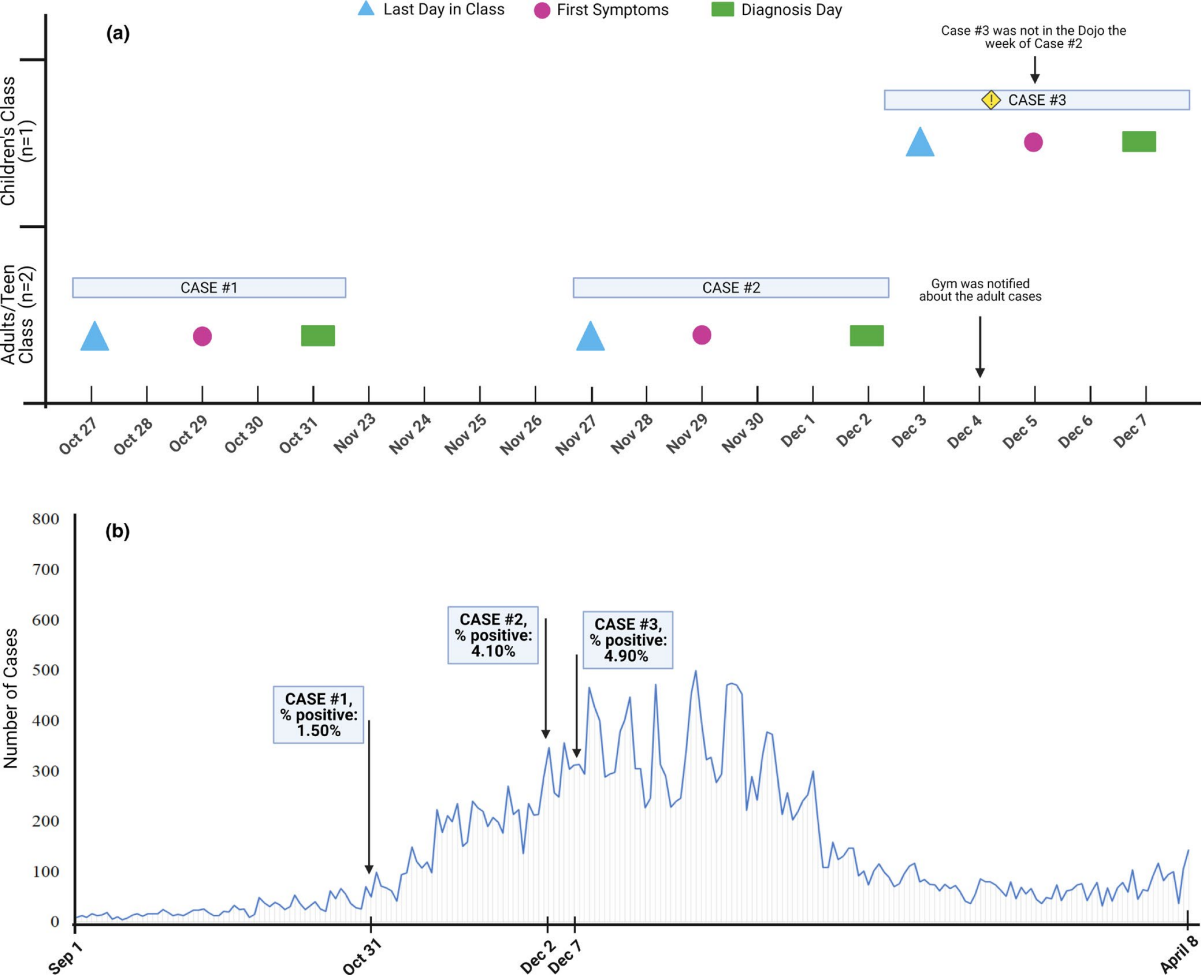
The timeline (a) of case 1 (teen; age:17), case 2 (adult; age: 48) and case 3 (child; age: 6) and the total number of cases/day (b) for the county during the period of the case reports. (a) The last day in the Dojo (triangle), date of symptoms (circle) and date of the positive COVID‐19 test result (rectangle) are detailed from October 27‐December 7. Case 3 was not present in the Dojo the week of Case 2 and children's classes are earlier in the day compared to adult classes. Therefore, Case 2, and the air that Case 2 contributed to in the environment, did not have contact with Case 3. (b) The total number of cases/day and the percent positive on the day of each case are detailed from September 2020‐April 2021

This case report involves a Dojo in Manlius, NY over the time period of October 2020–December 2020, where intense exercise and shouting occur during each class. Of note, COVID‐19 vaccines authorized for emergency use (Pfizer‐BioNTech, Moderna, and Johnson and Johnson) were not available during this time period. New York State and Onondaga County had specific safety protocols that were required for businesses to operate. Therefore, the protocols were adhered to during the period of the cases and were also documented by video and personal observations of the corresponding author. Over a period of 2 months, three separate individuals (two adults and one child) who attended classes later tested positive for COVID‐19 (Figure 1a). Once a positive case was determined by the county, the Dojo was contacted. COVID‐19 cases by report date in Onondaga county over this time period shows a trend of substantial increase in the number of positive cases (Figure 1b). Antigen tests were the primary COVID‐19 test used in the county with nasopharyngeal or nasal swab specimens. There were two additional testing sites in the county that used a PCR test. The testing of employees was performed only after a positive case report. The county also had testing at multiple mobile sites throughout the community, a primary testing site at the convention center, asymptomatic testing at all schools, and many health care facilities and other businesses required routine testing. It is likely that other participants were tested based on the county surveillance testing. However, we did not measure this routine testing in our study, which is why we cannot say exactly how many other participants were tested and when.

**FIGURE 2:**
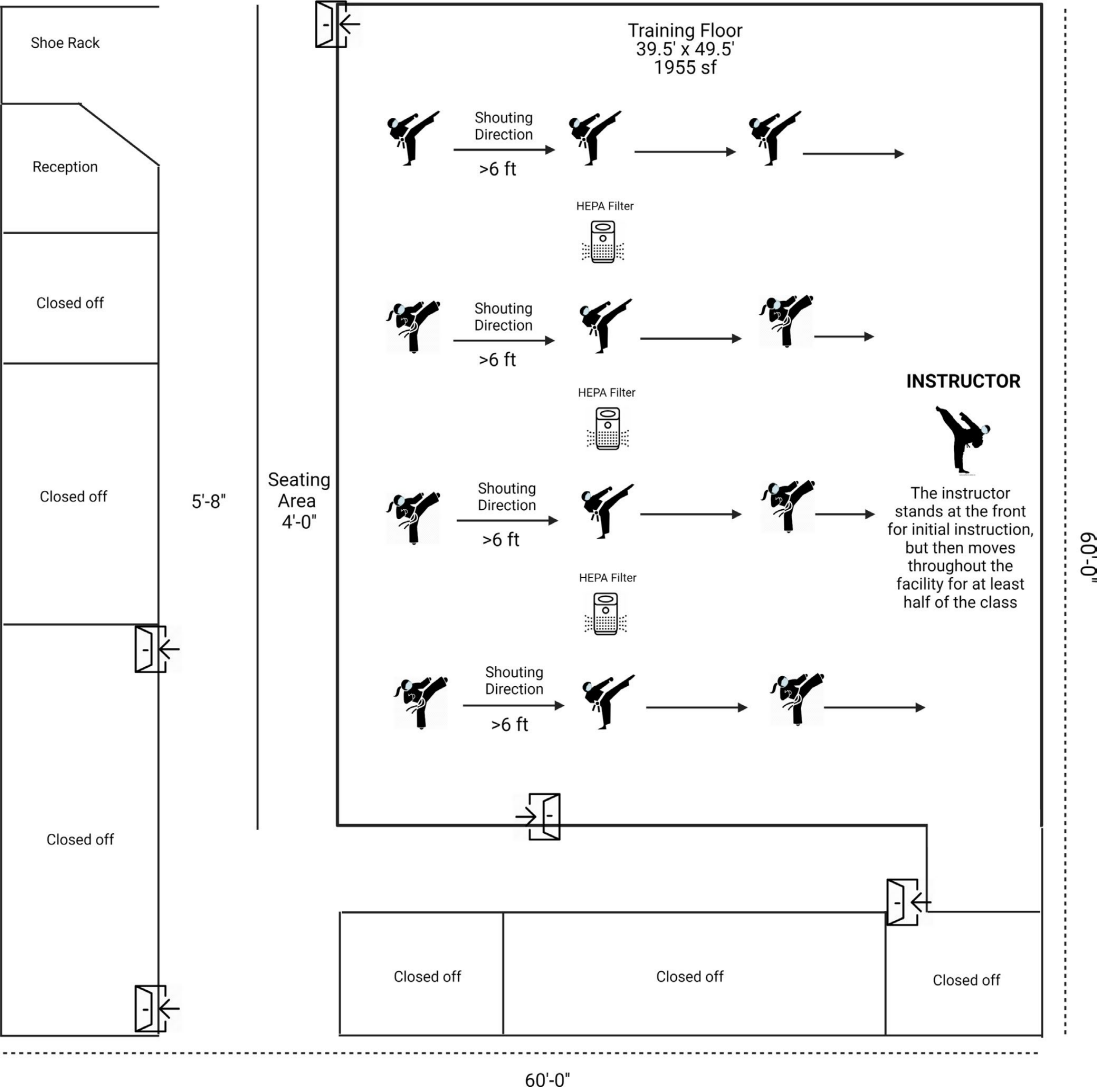
Training area included no more than 12 participants plus 1 instructor, all wearing a mask and keeping social distance >6 feet. High‐efficiency particulate air (HEPA) filters were on 30 min prior to practice and continued for the entire class period

The participants consisted of two adults and one child. The training area (1955 sq ft) is presented in Figure 2. The Institutional Review Board (IRB) at Le Moyne College deemed this project as exempt from IRB regulation. We still followed all precautions to de‐identify any personal data used in the case study. After each reported case, the Dojo was closed for cleaning and testing of all employees. While cleaning of surfaces was a common safety protocol practice in 2020, a recent report from the Centers for Disease Control and Prevention (CDC) CDC revealed that the transmission risk from surfaces is relatively low compared to airborne transmission of the virus. Indeed, a recent review from Czypionka et al. (2021) demonstrated a stronger chance of airborne transmission of SARS COV‐2 in indoor environments through aerosols when an infected person exhales, speaks, shouts, sings, sneezes, or coughs. To the best of our knowledge, no other known spreading of the virus occurred to any individuals following each of the positive cases, demonstrating the safety protocols (particularly those preventing airborne transmission of the virus) taken by the exercise facility were effective and/or the individuals were not infectious during the training. It is important to note, however, the virus transmission (due to asymptomatic spread) still could have occurred outside of our knowledge. Here, we lay out the safety protocols followed in these real‐life instances of COVID‐19 positive cases in an exercise facility.

## 3 SAFETY PROTOCOLS IN EXERCISE FACILITY

- At the entrance, body temperature was checked, and hand sanitizer was used for each participant.
- Three portable HEPA filters (WAGNER Switzerland, Model: WA‐777, Voltage: 100–120V.50/60 HZ, Cleaning time and area: Cleans air in 10‐minutes and/or 5 air changes/hour in rooms up to 500 square feet. Power Consumption: 50W, Noise Level =L 21‐41 db, China) were utilized and turned on 30 min prior to each class (Figure 2). No windows or doors were open, although the heat was typically on throughout the time period described (thermostat set to 68–72ºF).
- HEPA filters were changed every 6 months for proper maintenance of air filtering.
- Duration of classes were reduced from 45–60 min to 30 min.
- The maximum number of participants in a class was reduced from 40 to 12. Two employees were present during the case report for most classes, but only one instructor was on the floor at a given time. Previously, unlimited observers were allowed with each student. However, during the case report time period, each student was allowed only one observer. In reality, there was zero or one observer present in the Dojo for any given class. Most parents/guardians/observers waited outside or in their cars.
- Face coverings (homemade or surgical masks) without exhalation valves were required at all times.
- Social distancing was always maintained with participants at least 6 feet apart with rectangular markings on the floor for each person to stay within their area.
- All floors in the facility were swept, mopped, and disinfected daily.
- All counter tops, bathrooms, and drinking fountains in the facility were cleaned and disinfected daily.
- All touch surfaces in the facility were sanitized in between each class.
- Changing rooms in the facility were closed and bathrooms were allowed to use on urgent basis only.
- Digital sign‐up was required for each class for contact tracing.
- Videos of each class were recorded if additional contact tracing was needed.
- Employees were required to fill out a questionnaire regarding their overall health each day.
- One parent or guardian per participant was allowed inside the Dojo during the 30‐minute class period, although most parents waited outside the facility.

## 4 DISCUSSION

The magnitude of virus spread in exercise facilities may stem from many factors including the number of participants, the size of the facility used specifically for training, and the intensity of exercise (Klompas et al., 2020). Other than the factors associated with an exercise facility, latitude, wind speed, and socioeconomic status also influence the transmission rate of the virus (Cao et al., 2021). Additionally, the % positive (i.e., the percentage of all COVID‐19 tests performed that are positive) in the community contributes to overall risk. The large volume of air flow with virus‐containing respiratory droplets from individuals exercising at a high intensity may be particularly problematic when combined with poor ventilation in a crowded small place where social distancing is difficult (Klompas et al., 2020). However, we report no known viral spread when masks, social distancing, and HEPA ventilation were utilized in a high intensity exercising environment with shouting/loudness of vocalization, which is associated with rapid spread of COVID‐19 (Hamner et al., 2020). For martial artists, shouting is a manifestation of fighting spirit and the internal desire to prevail under difficult situations (Cynarski et al., 2019) and is also a primary component to the training. Of note, the % positive at the time of the case studies was steadily increasing in the county (case 1 = 1.50%, case 2 = 4.10%, and case 3 = 4.90%; Figure 1b), demonstrating an increased risk compared to earlier time periods when no cases were detected in the Dojo. Although there is no established information about what is a “low” percent positive rate, anything ≥5% is considered “too high” (Dowdy & D’Souza, 2020).

Many countries imposed strict measures in enclosed areas, including exercise facilities to prevent the rapid spread of COVID‐19. However, the current literature indicates that improving and maintaining physical fitness are crucial for a better COVID‐19 prognosis. A recent study from Brawner et al., (2021) showed that aerobic fitness (as quantified by the metabolic equivalents of task (METs)) is an inverse and independent predictor of COVID‐19 hospitalization in infected individuals. Regular exercise has a variety of other immunological benefits that can prevent hospitalization and death rates associated with COVID‐19 (Simpson & Katsanis, 2020). Data from epidemiological studies suggest that regular exercise can protect the host organism from infections similar to COVID‐19 such as influenza, rhinovirus, Epstein–Barr, varicella zoster, and herpes simplex virus 1 (Duggal et al., 2019; Simpson & Katsanis, 2020). Individuals with a regular exercise habit also report fewer symptoms associated with upper respiratory illnesses, common manifestations of COVID‐19 (Martin et al., 2009; Simpson & Katsanis, 2020). While exercise is known to be protective for other respiratory illnesses, the ability to attend community‐based exercise programs was stopped during lockdowns for the safety of the overarching community.

In order for closed environment exercise facilities to re‐open, strict safety protocols were followed in New York State. According to John Hopkins University Schools of Medicine Coronavirus resource center, the percent positive rate of 2.8% in New York State was substantially lower than the average percent positive of 8.3% in the USA between October 27 and December 7, the time period the cases in our study were identified. The same source shows, as of May 25, 2021, the New York State has a percent positive of 0.9%, whereas this percent rate is 2.5% for the country. However, data regarding positive cases identified in exercise spaces with safety protocols and possible viral spread are limited. Here we report no symptomatic spread of COVID‐19 in a closed environment with universal mask wearing, HEPA filtration, and social distancing. In all three cases, the participant was in the facility 2 days prior to symptoms. Importantly, the viral load of SARS‐CoV‐2 peaks around 1–2 days before symptom onset (Cevik et al., 2020), although we did not measure the viral load of each positive case. In this small sample, these findings indicate indoor, high intensity exercise with shouting under the specified conditions did not promote symptomatic spread of COVID‐19. As more exercise facilities open to full capacity, understanding (and following) the safety protocols that prevent possible spread of COVID‐19 will be useful for the safety of the community. Of note, the cases reported in our case study were detected before the recently observed variants of SARS‐CoV‐2 were wide spread. Therefore, it is unclear how these safety protocols will work specifically for the new variants.

## CONFLICT OF INTEREST

The authors declare no conflict of interest.
